# Rapid assessment of fine needle aspiration and the final diagnosis – how often and why the diagnoses are changed

**DOI:** 10.1186/1742-6413-3-25

**Published:** 2006-11-06

**Authors:** Carolyn Woon, Ricardo H Bardales, Michael W Stanley, Edward B Stelow

**Affiliations:** 1 Department of Pathology and Laboratory Medicine, University of Minnesota, Minneapolis, MN, USA; 2 Outpatient Pathology Associates, Sacramento, CA, USA; 3 Hospital Pathology Associates, St. Paul, MN, USA; 4 Department of Pathology, University of Virginia, Charlottesville, VA, USA

## Abstract

**Background:**

On-site rapid interpretation (RI) of fine needle aspiration (FNA) has been shown to increase the diagnostic yield of FNA and decrease the need for repeat diagnostic procedures. Because the pathologist interprets only a fraction of the sample and has limited resources available at such times, an occasional RI diagnosis will be changed at the time of the final diagnosis. We investigated how often these changes in diagnoses occur and the possible reasons for the changes.

**Methods:**

All cytology reports from 1/1/02 to 12/31/03 from a single institution were reviewed. Cases with RI with discrepant final diagnoses were noted. The discrepant diagnoses were categorized depending on how they were changed. Possible sources for changed diagnoses were noted.

**Results:**

Between 1/1/02 and 12/31/03 there were 1368 RIs of FNAs. Of these 80 (5.8%) had discrepancies between the RIs and final diagnoses. Seventy-eight cases had additional slides and/or cell block at time of final diagnosis. 16 cases had ancillary studies available at final diagnosis. Consultant pathologists were used in 7 cases. Different pathologists interpreted the RI and final diagnosis in 31 cases.

**Conclusion:**

Although uncommon, discrepancies between RIs and final diagnoses occur 5.8% of the time at our institution. Most commonly, this involves a change of diagnosis from either "non-diagnostic" or "benign" to "malignancy". Although much of this is likely due to the presence of additional material and information at the time of final diagnosis, the number of cases that had different pathologists involved in the RI and final diagnosis suggests that inter-observer variability may also play some role.

This data was originally presented at the 2005 meeting of the United States and Canadian Academy of Pathology in San Antonio, Texas, March, 2005.

## Background

On-site rapid interpretation (RI) of fine needle aspiration (FNA) has been shown to increase the diagnostic yield of FNA and decrease the need for repeat diagnostic procedures. Previous studies have shown that up to 32% of FNAs performed without on-site rapid interpretation are non-diagnostic [1-12]. On-site evaluation of FNA specimens can be beneficial in determining adequacy, triaging of specimens for ancillary studies as well as providing a preliminary diagnosis to the clinicians for possible rapid clinical decision making [12]. Because the pathologist interprets only a fraction of the sample obtained and has limited resources available at such times, an occasional RI diagnosis will be changed at the time of the final diagnosis. Furthermore, inter-observer variability may play some role in these changes as different pathologists may sometimes review the on-site material and the final material. We investigated at our institution how often these changes in interpretations occur as well as the reasons for the changes.

## Materials and methods

All cytology reports from 1/1/02 to 12/31/03 from a single institution were reviewed for FNA cases in which an on-site interpretation (RI) was performed. The aspirates were performed by the clinician, radiologist or pathologist. All on-site evaluations, including the preparation of smears, rendering of preliminary diagnosis and triage of specimen for possible ancillary studies were performed by an attending pathologist (RHB, MWS and 2 others not included as authors). Of these pathologists, 2 of 4 (RHB and MWS) had subspecialty training in cytopathology. In all cases, at least one air-dried, Diff-Quik^® ^stained smear was prepared for immediate microscopic evaluation. Additional smears were fixed in alcohol and stained with the Papanicolaou stain and reviewed at final interpretation. If additional material was available, cell blocks were prepared by allowing material to clot, scraping it into 10% buffered formalin and then processing the material by routine histologic methods. Ancillary studies were submitted at the discretion of the pathologist who interpreted the material on-site. Final interpretation included the interpretation of all ancillary testing.

The diagnosis rendered during RI was compared to the final diagnosis. Cases with discrepant diagnoses were categorized depending on how they were changed. Possible sources for changed diagnoses included additional smears and cell block, the use of ancillary techniques such as immunohistochemistry and flow cytometry, the use of consultant pathologists, and changes in the pathologist on service at the time of RI versus final diagnosis. All diagnoses (RI and Final) were categorized as either non-diagnostic (ND), benign non-neoplastic (BNonN), benign neoplasm (BN), atypical/suspicious (AS) or malignant (M).

## Results

Between 1/1/02 and 12/31/03 there were 1368 RIs of FNAs. Of the 1368 RIs, 80 (5.8%) had discrepancies between the RIs and final diagnoses (see table 1). Of the 80 cases with discrepant RI and final diagnoses, eleven were considered to be false positives (10 cases originally interpreted as A/S and 1 originally interpreted as M). Specimens originally deemed ND were found to be BNonN (10), BN (3), AS (4), and M (21). Specimens deemed BNonN were found to be a different BNonN (7), BN (2), AS (4), and M (12). Specimens deemed BN were found to be BNonN (2). Specimens deemed AS were found to be BNonN (10). A single case deemed M was found to be BNonN. Four cases deemed M were found to be different Ms. Seventy-eight cases had additional slides and/or cell block at time of final diagnosis. Sixteen cases had ancillary studies available at final diagnosis. Consultant pathologists were used in 7 cases. Different pathologists interpreted the RI and final diagnosis in 31 cases.

**Table 1:** Discrepant Diagnoses between Rapid Interpretations and Final Diagnoses

**On-site Interpretation**	**Final Diagnosis**
Non-diagnostic (38)	Benign, non-neoplastic (10)
	Benign neoplasm (3)
	Atypical/suspicious (4)
	Malignancy (21)
Benign, non-neoplastic (25)	Different benign non-neoplastic (7)
	Benign neoplasm (2)
	Atypical/suspicious (4)
	Malignancy (12)
Benign, neoplasm (2)	Benign. Non-neoplastic (2)
Atypical/suspicious (10)	Benign, non-neoplastic (10)
Malignancy (5)	Benign, non-neoplastic (1)
	Different malignancy (4)

## Discussion

The use of FNA biopsy has become an important diagnostic tool for the work-up of the clinical patient. It is a highly safe and accurate technique causing minimal discomfort, trauma and complications to the patient. Though not uniformly practiced in all centers, an on-site RI of the FNA is often performed. This has been shown to increase the diagnostic yield of the aspirate as well as minimizing the incidence of repeat biopsies [3-5,7,17-19]. The main indication of an on-site RI of the fine-needle aspirate is to determine the adequacy of the specimen for definitive evaluation, thus minimizing the unsatisfactory rate [14]. Based on the available material, a specific preliminary diagnosis can often be rendered by the examining pathologist, guiding further clinical investigations and decisions, as well as determining the need for further ancillary studies including flow cytometry, microbiology cultures, molecular studies and immunohistochemistry.

While the performance of the FNA biopsy has increased markedly, there have only been a handful of reports looking at the discrepancy rate between the RI diagnosis and the final diagnosis. As far as we know, our study is one of the largest of its kind with a total of 1368 cases of FNAs with on-site RI obtained over a two year period. Interestingly, a major proportion of our RIs occurred with specimens obtained via endoscopic-ultrasound guided FNA.

In our study, we found that the diagnostic discordance rate was approximately 5.8% with 80 cases for which the RI diagnosis was different from that of the final diagnosis. These results are similar to previously published studies that report concordance rates ranging from 82% to 100% [5-7,12-16]. Of the 80 cases, there were 41 false negative diagnoses (if one includes cases originally interpreted as non-diagnostic) and 11 false positive diagnoses giving a false negative and false positive rate of 3.00% and 0.80%, respectively.

Our review of all 80 RI diagnosis cases with discordant final diagnoses suggests that the major cause of discrepancy occurred secondary to sampling errors in which diagnostic material was not present on the originally interpreted slides, but was present in the additional material interpreted at sign-out (alcohol fixed slides/cell block). This error likely accounted for the majority of false negative diagnoses rendered in our institution (38 cases found to be ND at RI had a diagnosis at sign out). This is not surprising given the fact that the pathologist is only able to examine about half of the submitted sample on-site (at our institution), which may or may not include adequate diagnostic material. The remainder of the submitted specimen is fixed in alcohol or prepared for cell block and was only available for examination the next working day. Furthermore, we believe that, in general, we set our diagnostic threshold somewhat higher during RI, hoping to obtain more material and thus eliminating final unsatisfactory diagnoses. Other causes of the discrepancies include the eventual availability of additional ancillary studies and additional clinical history at time of final diagnosis. Interpretive errors constituted a very small percentage in the cause of discrepancy during on-site RI. These were predominately the false positive diagnoses.

Out of the 80 cases of discrepant diagnoses, we identified only eleven false positive cases for which the RI diagnosis was deemed atypical/suspicious (10) or malignant (1), but was found to be benign non-neoplastic at final diagnosis. These errors could be considered clinically significant as they may lead to an unnecessary surgical or medical treatment. Because we know that these errors can and do occur, we do try to stress the fact that RIs are preliminary interpretations and that treatment should not be instituted based on these diagnoses, especially with atypical/suspicious diagnoses.

Interestingly, at our institution, 31 of the 80 (38.8%) discrepant diagnoses involved different pathologists evaluating the RI and the final diagnosis. Given the fact that our pathologists generally rotate on a weekly schedule, this suggests that inter-observer variability may also play some role in contributing to the discrepant diagnosis. Further studies need to be done to investigate the extent this factor contributed to the incidence of discrepant diagnoses.

In conclusion, our study confirms that the diagnoses rendered during RI are highly accurate and are usually concordant with the final diagnoses. However, our study shows a 5.8% discrepancy rate between RIs and final diagnoses. With this in mind, pathologists should communicate to clinicians the limitations of the RI, and clinicians should in turn delay any unnecessary critical decisions regarding therapy until the final diagnosis becomes available.

## Competing interests

The author(s) declare that they have no competing interests.

## Authors' contributions

CW participated in data acquisition, analysis, and the writing of the study.

MWS participated in the coordination of the study.

RHB participated in the coordination of the study.

EBS conceived of the study, participated in data acquisition, analysis and the writing of the study
